# Delta-P as an early indicator of oxygenator failure: the case for standardized manufacturer reference values

**DOI:** 10.1051/ject/2025039

**Published:** 2025-12-17

**Authors:** Salman Pervaiz Butt, Salman Abdulaziz, Ashwaq Mahdaly, Nabeel Razzaq, Drisya Paul, Umer Darr, Gopal Bhatnagar

**Affiliations:** 1 Perfusion Services, Heart Vascular and Thoracic Institute, Cleveland Clinic PO Box 112412 Abu Dhabi United Arab Emirates; 2 Critical Care Services Administration, As Sulimaniyah Makkah rd Riyadh 12231 Saudi Arabia; 3 Department of Critical Care, As Sulimaniyah Makkah rd Riyadh 12231 Saudi Arabia; 4 Heart Vascular and Thoracic Institute, Cleveland Clinic PO Box 112412 Abu Dhabi United Arab Emirates

**Keywords:** Delta-P monitoring, Oxygenator thrombosis, ECMO circuit safety

## Abstract

The evolution of extracorporeal membrane oxygenation (ECMO) has led to an increasing reliance on objective parameters to detect complications early and enhance patient safety. One such parameter is the transmembrane pressure gradient, more commonly referred to as delta P (ΔP), the difference between the inlet and outlet pressures of the oxygenator. This measure has increasingly been recognized as a critical early indicator of thrombus formation within the oxygenator during ECMO support. However, its interpretation remains complex and context-dependent, particularly because delta P values are significantly influenced by the design features of different ECMO circuits, including pump head geometry and oxygenator configuration.

## Discussion

Thrombus formation in the ECMO oxygenator poses a significant threat to circuit integrity and patient safety, often necessitating emergent oxygenator exchange. Early recognition can be lifesaving, and delta P has become a widely used, non-invasive monitoring surrogate for potential clot burden within the oxygenator. Yet, there is no universal threshold for “critical” delta P because baseline values and progression trends differ across manufacturers and models. This variability often leads to ambiguity in clinical decision-making, particularly in high-volume ECMO centers where multiple oxygenator brands and circuit types are in use simultaneously.

The pump head size and flow characteristics, membrane surface area, internal resistance to flow, and even the pressure measurement points incorporated by the manufacturers all contribute to differences in baseline and dynamic delta P readings. For instance, some oxygenators exhibit naturally higher resistance due to their compact design or due to fiber density, thereby demonstrating a higher “normal” delta P at baseline compared to larger-surface oxygenators that permit higher flow at lower pressures. Furthermore, oxygenators that incorporate pressure sensors at different points within the circuit tubing or housing can yield differing readings even under identical flow and viscosity conditions [1].

In addition, the type of ECMO, venovenous (VV) or veno-arterial (VA), and the operational flow ranges will also impact delta P. In pediatric and neonatal circuits, for example, where flow is low and circuit components are small, minor fluctuations in delta P could indicate early clot formation. Conversely, in adult VA-ECMO systems running high flows, gradual rises in delta P might be attributed to progressive membrane fouling or hemoconcentration rather than acute clot formation, unless interpreted within the context of the specific oxygenator’s normal resistance profile.

Given these inter-manufacturer and model-specific variations, it is becoming increasingly important for ECMO teams to have access to standardized delta P reference curves provided by the manufacturers. These should include expected delta P ranges across various flow rates and blood viscosities under non-thrombogenic conditions. Having these benchmarks readily available would enable clinicians to detect deviations from expected resistance patterns more accurately and take preemptive action.

Such standardized data would be particularly valuable for high-volume ECMO centers that use multiple ECMO platforms and circuit configurations in parallel, driven by clinical demands, vendor availability, or institutional procurement policies. In these environments, bedside teams, perfusionists, and ECMO specialists frequently encounter the challenge of interpreting delta P trends across different equipments. In the absence of reference values, there is a risk of either delaying oxygenator replacement, potentially leading to hemolysis, embolic events, or oxygenator failure, or prematurely changing it, thereby increasing costs and exposing patients to additional circuit-related risks [2].

Providing this data would not only enhance patient safety and operational efficiency but also facilitate training, research, and benchmarking across institutions. Standardized delta P data would enable clinicians to develop predictive algorithms, contribute to multicenter registries, and establish early warning systems for oxygenator failure. Moreover, as ECMO centers move toward integrating machine learning and smart monitoring systems, access to manufacturer-specific delta P norms will be essential for model training and anomaly detection [3].

We, therefore, advocate for a collaborative initiative between ECMO manufacturers, regulatory bodies, and clinical societies to establish and disseminate reference delta P values for each oxygenator and pump combination under typical flow and hematocrit conditions. This information should be made easily accessible, potentially embedded into user manuals, clinical apps, or included in the graphical user interfaces of ECMO consoles.

As the clinical use of ECMO continues to expand across diverse patient populations and healthcare settings, the need for standardized, manufacturer-specific delta P reference data has become increasingly evident. Variability in oxygenator design, flow dynamics, and pressure measurement protocols contributes to significant heterogeneity in baseline and dynamic transmembrane pressure gradients, complicating clinical interpretation. The provision of validated delta P reference ranges, stratified by flow rate, hematocrit, and circuit configuration, would facilitate earlier detection of oxygenator dysfunction, reduce unnecessary circuit changes, and support the development of predictive monitoring algorithms. We advocate for a coordinated effort among industry partners, regulatory agencies, and clinical societies to generate and integrate these reference standards into both educational resources and ECMO system interfaces, thereby promoting safer, evidence-based practice.
